# Championing women working in health across regional and rural Australia – a new dual-mentorship model

**DOI:** 10.1186/s12909-020-02219-w

**Published:** 2020-09-11

**Authors:** Teresa M. Wozniak, Esther Miller, Kevin J. Williams, Amelia Pickering

**Affiliations:** Menzies School of Health Research, Charles Darwin University, Darwin, Northern Territory Australia

**Keywords:** Mentorship, Resource poor settings, Workforce retainment, Professional development, Evaluation

## Abstract

**Background:**

Mentoring is a critical component of career development and job satisfaction leading to a healthier workforce and more productive outputs. However, there are limited data on mentorship models in regional areas and in particular for women aspiring to leadership positions. Mentorship programs that leverage off experienced mentors from diverse disciplines have the potential to foster the transfer of knowledge and to positively influence job satisfaction and build capacity within the context of workforce shortage.

**Methods:**

This study describes a dual-mentorship model of professional development for women working in health in regional and rural Australia. We present the framework and describe the evaluation findings from a 12-month pilot program.

**Results:**

Both academic and corporate mentors provided diverse perspectives to the mentees during the 12-month period. On average, corporate mentors met with mentees more often, and focused these discussions on strategy and leadership skills whilst academic mentors provided more technical advice regarding academic growth. Mentees reported an improvement in workplace interconnectedness and confidence at the completion of the program.

**Conclusion:**

We developed a framework for establishing a professional mentorship program that matches women working in regional health with mentors from diverse sectors including business, government, philanthropy and health, to provide a holistic approach to improving career satisfaction, institutional productivity and supporting a diverse workforce in regional or resource-poor settings.

## Background

Despite the high overall health status of many Australians, rural and regional areas within this country continue to experience comparatively poor health outcomes [1]. Health inequity is a complex issue which is complicated by workforce shortages [2, 3] and high turnover of staff in regional and remote parts of Australia [4]. While retention of health professionals is a complex issue [5], initiatives to recruit and retain regional and remote staff have traditionally focused on financial reimbursement [6–8]. Far less attention has been paid to the benefits of professional networks and formalized mentorship programs in regional settings [9].

Literature shows that professional networks and formalized mentoring programs provide an opportunity to exchange knowledge, develop leadership skills and advance careers [10]. Mentorship can improve mentee productivity, self-efficacy and career satisfaction [11–14] and be a personally fulfilling experience for mentors [15, 16]. Well-structured and inclusive mentoring programs are highly beneficial for health professionals, including training researchers across varied disciplines [12, 17–24]. Mentoring relationships that offer the greatest impact on success are likely to be influenced by individual attributes [25] and the social context within which the program is instituted [26]. Mentoring can be didactic [27], peer-peer [28], formal or informal, delivered face to face or distance [29].

It is widely recognized that career advancement in medicine, research and health more broadly, remains in favor of men [30]. Not only do women receive less mentoring than men [31, 32], they are under-represented in senior roles [30], continue to be asked about their job commitment [33, 34], and get paid less. While women comprise roughly 47% of all employees in Australia, they take home $242.90 less than men each week (in November 2019) and the national gender “pay gap” has remained around 15–20% for most of the past two decades [35].

We sought to identify the needs of women working in the geographically isolated north of Australia and used these to inform the key elements of a new dual-mentor model, called the Catalyse Mentorship Program (“Program”) for women working in healthcare within a resource-poor setting.

## Methods

### Establishing networks and need for mentorship program

In 2018, a professional network called Women in Tropical Health (WITH) was established as part of a large health research program, ‘Improving Health Outcomes of Northern Australia’ (HOT North). In November 2018, a needs analysis survey was conducted to determine the requirement for a formalized mentoring program in regional and rural Australia (supplement Table S1). Within the scope of the WITH network was the pilot and a formative evaluation of the Catalyse Mentorship Program (the ‘Program’) (Table 1).

**Table 1:**
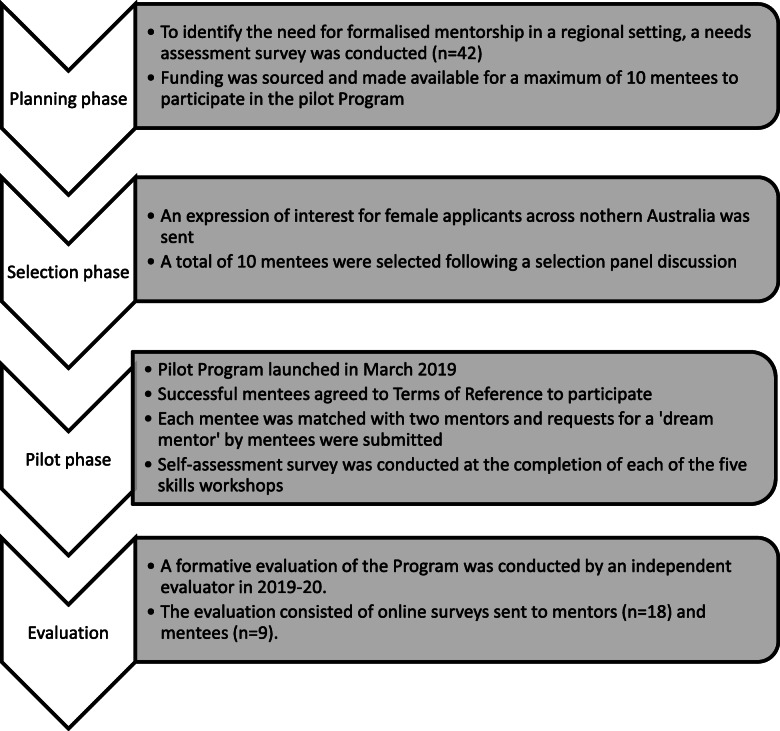
A phased approach to establishing and evaluating the Catalyse Mentorship Program

### Catalyse Mentorship Program (“Program”) structure

The primary aim of the Program is to facilitate diverse partnerships which provide women with opportunities to reflect on and grow their leadership capabilities, build professional networks, and more effectively navigate career progression and development .

There are four main objectives:

1. To provide a safe environment where professional knowledge, experience and advice is shared between and within mentees and the Program facilitator
2. To cultivate a strategic approach to career planning and to foster innovation
3. To expose mentees to alternative career pathways and opportunities to consider advancing their work
4. To improve visibility of the mentee and their research

The Program employs a dual-mentor design that is concentrated on matching a female healthcare professional with an academic and corporate mentor, supported by facilitated mentoring resources and career-development workshops (Table S2). The dual-mentor model is designed to not only provide guidance on a broader range of skills, but also to introduce women to potential external funders, influential supporters and strategic counsellors and mentors (corporate, philanthropic and academic) who may be able to assist in advancing their work interests and careers. In the pilot Program, a AUD $2000 bursary was offered to each mentee to be used on professional development activities.

Mentees were expected to meet with both mentors four times throughout the Program, as well as attend five workshops. As part of the workshop resource packs, participants were provided with a meeting framework, with objectives and discussion points for each meeting. The strategic framework is described in Table 2.

**Table 2:** Strategic framework and potential outcomes of the Catalyse Mentorship Program, Australia 2019–2020

Objectives	Catalyse Mentorship Program activities	Outcomes and potential impact
1.To provide a safe environment where professional knowledge, experience and advice is shared between and within mentees and Program facilitator	•Five workshops with Program facilitator – offered face to face or virtually for those in remote regions (Supplement Table S2) •Rules of engagement established and directed by the group (Workshop 1) •Clear articulation of expectations and boundaries for mentees and mentors •Network opportunities for mentees and mentors •One-on-one meet ups and progress tracking with Program facilitator and mentees at Workshop 4 •Structured (and protected) time for personal and professional evaluation and identification of strategic next steps •Cultivate mentee and mentor relationships though regular ‘check ins’	•Peer-to-peer engagement and encouragement •Collaborations between mentees and institutions •Early establishment of peer-to-peer and mentor-mentee trust •An inclusive and supportive culture •Intentional and strategic career decisions •Wider and greater self-awareness
2. To cultivate a strategic approach to career planning and to foster innovation	•Emotional Quotient and Social Skills Training (Workshop 2) •Identifying roadblocks and challenges (Workshop 2) •SMART goals, action plans and resource acquisition (Workshop 3) •Intentional continuous personal development cycle (Workshop 5) •The art of asking – others for what you need for your professional develop (i.e. networking, funding or other types of support). •At the final workshop (Workshop 5), mentees would reflect on, and review bios and mission statements drafted in the first workshop and modify these if needed	•A more motivated and inspired workforce •Increased confidence •Professional and personal (i.e. self-care) intentionality •Potential for career advancement
3. To expose mentees to alternative career pathways and opportunities to consider advancing their work	•Successful matching of mentees with an academic mentor and a corporate mentor •Each mentee could apply to be matched with a ‘dream mentor’, connections were made where possible •How to have effective conversations (Workshop 2) •Mentor training and resourcing including mentor pack provided by Program facilitator •Each mentee was awarded a bursary to use of professional development opportunities	•Development of management and leadership skills set •Expansion of professional networks •Broadened career options •Exposure to potential funders and influential supporters
4. To improve visibility of the mentee and their research	•Developing a personal mission statement (Workshop 1 and review Workshop 5) and narrative about your professional self •Professional photography of mentee and LinkedIn profile created	•Potential for career advancement

### Program pilot – 2019/20

The Program pilot period was March 2019 until March 2020. During this period, each of the mentees were assigned two mentors; one academic mentor and one corporate mentor, recognizing that both mentor groups would bring specific skills and knowledge to the Program. This unique feature of the Program originated from a qualitative need’s assessment which identified diversity of expertise and networks as a challenge for women working in regional Australia.

The mentees were members of the WITH network and included healthcare professionals from across multiple institutions, including Menzies School of Health Research, Charles Darwin University, Flinders University and Torres and Cape Hospital Health Services.

Academic mentors were either known contacts of the Program management team who had previously expressed an interest in mentoring early/mid-career women or were identified by the team as possessing the skills and attributes which would lend them to be appropriate mentors in this Program.

Corporate mentors were selected from a pool of industry supporters and ambassadors cultivated by the Menzies Development Team over several years. A few however were recruited specifically for the Program due to their mentoring experience and/or desired expertise.

Most mentors were located along the eastern seaboard of Australia and have extensive networks nationally. Therefore, these pairings aimed to further offer exposure, scope and relational opportunities beyond northern Australia, or the nature of which mentees may ordinarily not have access to. Although many had experience as mentors previously, mentor training and support was provided in the form of an initial resource pack including guidelines and expectations, on-going intermittent e-resources, regular ‘check-ins’ and access to a Mentor Helpline (Table 2). Furthermore, mentees were provided an opportunity to put forward a name of a ‘dream mentor’ i.e. someone they have researched and believe had valuable insights, experience or connections that would enhance their personal and professional progression. Of the nine mentees who completed the Program, five secured dream mentors, two were unsuccessful and matched with an alternative mentor, and two did not submit a request.

During the first of the five mentee training workshops (described in greater detail in Supplement Table S2), the participants collaborated to collectively articulate their shared expectations of the Program. These were summarized as:

- To be linked up to people who are further in their career for mentorship
- To have a toolkit to develop leadership skills
- To have considered with the support of the Program facilitator a 5–10-year career plan
- To decide the next career step and why
- To gain increased confidence
- To be able to promote myself and my work
- To gain a more strategic approach to career development and change direction if needed
- To have dedicated time to reflect on my career
- To develop relationships with those who have blazed the path before me

At each of the subsequent workshops, these expectations were reviewed, and mentees participated in a self-assessment survey to assess progress made towards these objectives, as well as to identify broader learnings (Table 2 and Table S2).

### Evaluation of program

A formative evaluation was conducted between November 2019 and May 2020 to ensure that the Program was achieving its aims, and to document necessary adjustments or improvements for future program development. The evaluation considered the suitability of the Program resources, the application and selection processes, the matching process, the program format and duration, and the inclusion of financial support. It also considered whether the Program met its objectives through achievement of short-term outcomes.

An online, anonymous survey was sent to each participant of the Program, which included nine mentees, nine academic mentors and nine corporate mentors (Supplement Table S3 and Table S4). The surveys did not include questions relating to demographic information or current workplaces, projects or other information that could be used by the evaluators to identify any of the respondents.

Data analysis was performed in Excel with differences expressed as the number and percentage of responses.

## Results

### Women working in regional settings want formalized mentoring programs that offer diverse perspectives

There were 42 responses provided to the need’s assessment survey conducted in 2018 and prior to starting the pilot Program (Table S1). Most of these respondents were women who reported being mid-career (*n* = 23, 55%), with 28% (*n* = 12) early career and 17% (*n* = 7) in established leadership roles. Despite 95% (*n* = 40) of survey participants requesting a formalized mentorship program, the majority did not have the opportunities to participate in one (*n* = 38, 90%). The majority (88%) considered mentoring as a discussion about personal and professional goals whilst others thought it as an opportunity to be coached on a particular task. When asked “*What main areas of development the participant would benefit from the most*”, the two main themes that emerged were “clear career pathways” and “diverse perspectives”.

### The process of self-matching mentee-mentor pairs was appropriate

The Program used a ‘self-matching’ process to pair up mentors with mentees. This process was recommended by corporate mentors based on their experience that appropriate matching which facilitates ‘good chemistry fit’ was critical to a productive pairing. To this end, both mentors and mentees had input into the matching process by providing feedback and preferencing their matches. Mentors and mentees were provided with a portfolio of possible matches and were asked to identify their three “top choices”. The mentor portfolio included professional bios, as well as a personal statement regarding “*What I can offer that is not in my bio*”. The mentee portfolio included the professional bio and mission statement compiled in workshop one (Table S2), as well as the mentees top three challenges that they would like to address with mentors’ support. Mentors and mentees used this information to do an informal personal evaluation to determine who they would be best suited with: mentors from the perspective of whose challenges they identify with and whose work they are interested in supporting; and mentees from the perspective of who portrayed aspirational attributes, skills and experience. Preferences were then collated and compared in order to identify obvious matches (i.e. where both the mentor and the mentee ranked each other as a ‘top choice’). All parties were given an opportunity to contact the Program facilitator at any stage of the process, should they believe the pairing unsuitable. One mentee reported being ‘disengaged’ with their academic mentor however she did not request any changes be made with this arrangement.

The formative evaluation found that the majority of mentors and mentees agreed or strongly agreed that the matching process used in the program was appropriate. Three mentees (37.5%) were neutral, as was one academic mentor and no participants disagreed with the process of self-matching.

### Catalyse mentoring program was composed of a diverse group of mentors and mentees

The 12-month pilot included nine mentees from northern Australia and one international mentee, with one mentee not completing the pilot (90% completion). Each mentee was matched with two mentors, five of which were ‘dream’ mentors. A total of 18 mentors were recruited to the Program. The mentees represented a diverse group of health professional (academic, non- academic) at various levels of seniority. The mentors represented males (17%, *n* = 4) and females (83%) from a range of sectors including health, academia, transport, banking and insurance.

### The mentees met with corporate mentors more often than with academic mentors and raised different topics

**Fig. 1:**
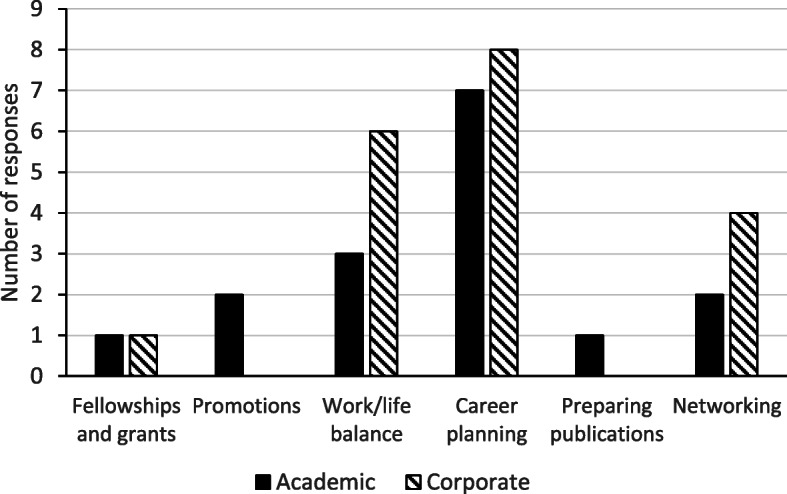
Topics discussed with academic and corporate mentors during Catalyse Mentorship Program pilot, Australia 2019-20

The evaluation survey showed that on average, mentees met with their academic mentors 3.25 times, and with their corporate mentors 4 times. During these meetings, academic mentors discussed fellowship applications, work/life balance and career planning, whilst corporate mentors mainly discussed the latter (Fig. 1).

Academic mentors provided specific ‘technical’ advice regarding the explicit and implicit academic growth pathway i.e. explicit pathways such as formal academic progression process and implicit avenues such as the advantages of connections, types of journals to publish in, and how to distinguish one’s specific work. Corporate mentors provided broad and specific objective advice on strategy, leadership and interpersonal skills. Specific examples include how to generate consensus within a team and with external stakeholders, how to have difficult conversations, how to build and express a personal brand in business.

### Mentees report an improvement in workplace interconnectedness, job satisfaction and confidence

**Fig. 2:**
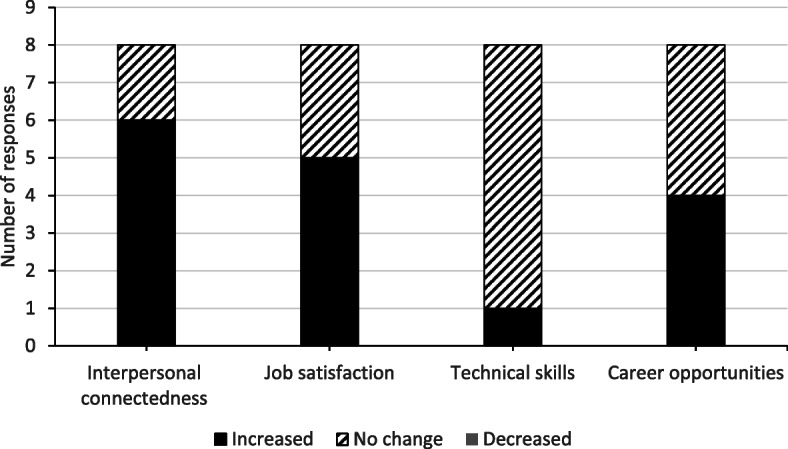
Short-term outcomes of the Catalyse Mentorship Program, Australia 2019–20

The evaluation questionnaire asked Program mentees to report any changes (increase, decrease or no change) to various measures of mentoring benefits during the pilot. Eighty percent of the mentees reported an increase in feeling of interpersonal connectedness in the workplace; and 62% (*n* = 5) reported an increase in job satisfaction. One mentee reported an increase in their technical skills (Fig. 2).

At the completion of the Program, 71% of the mentees self-assessed an increase in confidence and “*dedicated time to reflect on where things are and new goals to set”* (Table 3). Almost one third of the mentees reported having tools that can develop their leadership skills.

**Table 3:** Program expectations as reported by mentees “self-assessment survey”

Expectation of the Program, *N* = 7	Mentees who felt this expectation was met (%)
To gain increased confidence	71
To have dedicated time to reflect on my career	71
To be linked up to people who are further in their career for mentorship	57
To have considered with the support of Program facilitator a 5–10 year career plan	57
To decide the next career step and why	57
To gain a more strategic approach to career development and change direction if needed	57
To have a clearer understanding of where I want to go next	43
To be able to promote myself and my work	43
To develop relationships with those who have blazed the path before me	43
To have a toolkit to develop my leadership skills	29

### The perception of having achieved a set of program goals varied between mentees and mentors

As part of the Program workshops, mentees were asked to list three goals which they had hoped to achieve by the end of the program. The goals were categorized into three areas of focus: [1] ‘*Here and Now*’ e.g. manage workload, streamline tasks, repair relationships [2]; ‘*What’s Next*?’: e.g. actions needed to move towards the participants vision, upskill in certain areas, do investigations about options; and [3] ‘*Self-Care’*: e.g. identifying practises that can be incorporate into day-to-day life that will provide openings for transformation or creativity.

At the completion of the Program, the evaluation questionnaire asked both mentees and mentors to reflect on how well the mentee achieved these goals. Only one third (*n* = 3, 33%) of the mentees rated achieving this goal as very well or well, whilst the remainder were either neutral or poorly achieved. All the corporate mentors and 83% academic mentors felt that their mentees had achieved the goals which were set in the beginning of the Program.

### Mentee-self assessment survey report increased confidence and growth potential

Mentees describe how the Program has improved their relationship with their employing organization, by improving their strategic acumen, perspectives on organizational interdependencies and by reinforcing personal motivation and purpose. Furthermore, many mentees reference an increase in confidence, and as a result identifying or being identified as suitable candidates for greater or different career progression opportunities.

### “Incidental” outcomes of the program

The Program resulted in several promising and notable outcomes that were not initially intended. A summary of these is listed below

1. Additional ‘dream’ mentors secured to the Program. As quoted by a mentee when asked what unexpected outcomes they have experienced (self-assessment during a workshop) “*The fabulous mentorship relationships that I have developed with my two mentors*”.
2. A new peer-to-peer support group initiated and led by one of the Program mentees. As quoted by a mentee (self-assessment during a workshop) to be a highlight of the Program “*The wonderful support group that the participants created. I didn’t expect there would be so much laughter and jokes.*”
3. A new networking group established by a partnership from a Program mentee and corporate mentor
4. A new feasibility study conducted in collaboration with a Program mentee and corporate mentor to improve health service delivery in regional Australia
5. A business case to test the commercialization of a research Intellectual Property concept developed by Program mentee and corporate mentor for future career interests: *“Connection with the corporate mentor opened up opportunities I never would have explored by myself.”*
6. Prestigious academic awards and accolades
7. Philanthropic sponsorship to the Menzies School of Health Research from a Program corporate mentor, who had no connection to the organization prior to the Program.

## Discussion

We describe the successful pilot of the Catalyse Mentorship Program (the “Program”). A formal evaluation combined with self-reported surveys demonstrates that the Program resulted in the mentees experiencing positive ‘discomfort’, by being pushed out of their ‘comfort zones’ and requiring them to think differently. Many have changed their behaviors, as demonstrated by improving time management and priority-setting; and being “*more inclined to meet people and make the first move*”. This is further demonstrated through first-time activities undertaken by the mentees such as: business case development, establishment of peer-to-peer networking groups; application for (or nomination and receipt of) prestigious awards and accolades. For some, the Program has not had the same immediate affect *“I’m not as clear about my next step as I would like to be. But that’s ok.*” In this instance, it has provided these mentees a way forward through the “*tools I learned about in this program.*”

We found that within the regional context, relying on one style of mentoring (i.e. didactic or peer-peer support) may not be suitable. Traditional hierarchical mentoring with unidirectional mentor-mentee relationships have worked well in large research institutes and shown transformative mentorship for junior faculty in the short [36] and long-term [37]. However, these relationships in regional settings can be time-consuming and challenging when mentors are faced with demands of working multiple roles (clinical, research, teaching) and having overlapping roles as mentors and supervisors [38]. We were not able to identify published literature that describes a dual-mentor model (i.e. mentee is matched with an academic expert and a corporate mentor). However, there is strong evidence for the benefits of interdisciplinary perspective and cross-cultural mentorship [39–42]. Hence, choosing a mentorship program which emphasizes the reciprocity and co-learning of a mentoring experiences [27] adapted to local context was the model that we found most suitable. Linking across healthcare and industry sectors provides opportunities for a more diverse pool of mentors and mentees providing broader scope and exposure both vocationally and geographically, and a more inclusive and sustainable approach in the regional context.

Not all measures of a perceived successful mentorship program were achieved. We found a divergence in responses relating to achieving Specific, Measurable, Achievable, Relevant, Time-bound (SMART) goals set at the beginning of the Program. An explanation of this is likely multifactorial and may be a combination of recall bias (as formative evaluation survey asked to recall goals set more than 10 months prior) or differences in the interpretation of ‘achievement’ by mentees and mentors as it relates to goal progress [43]. To more clearly understand this, in-depth interviews would have been a more appropriate measure of assessing goal attainment in the Program. Assessing the impact of mentorship programs should ideally use a mixed methodology approaches. These include quantitative measures of “what” improved for participants and qualitative approaches that provide a deeper understanding of “how” programs work best.

The dual mentorship model as evidenced in the Program, provides an outstanding example of corporate female leadership to a cohort of women who are likely missing out on such opportunities. In the Program pilot, only 50% of the academic mentors were women, whilst all the corporate mentors were female. This may be a factor of a lack of female representation in senior academia [44] and more broadly across the global health sector [45]. The meetings with corporate mentors offered an alternative perspective. In this Program, academic mentors provided technical academic pathway advice, whereas meetings with corporate mentors focused more on interpersonal skills, teamwork and leadership pathways. Together, the dual mentorship model provides a rounded approach for mentees, offering both the discipline-based technical advice and external guidance on personal attributes and strategic thinking needed to lead.

The successful implementation of the Program does not automatically imply creating a mentorship culture but it’s a start. To leverage the gains from the Program pilot, mentees and mentors were asked if they would contribute in future programs and all agreed. Importantly, mentees expressed interest in participating in the next iteration of the Program in leadership roles, ranging from program facilitators, mentors or personal supporters. We believe that the Program has offered personal and professional opportunities to its participants to be both leaders and learners. It provides a framework for mentorship embedded into the institutional culture, which is suitable to the local context. We acknowledge that institutional support is a salient influencer of the success of formalized mentorship programs [46].

Though workforce retention was not an initial aim of the Catalyse Mentorship Program, we believe that providing strong professional networks in regional Australia may prove to be one initiative, in addition to others [47–50], to assist in the reduction of avoidable turnover. Regional Australia suffers from high staff turnover in healthcare [4]. Women who leave their place of employment or who are not able to contribute their full potential represent a loss of institutional return on investment [51]. For institutions, staff leaving creates gaps in leadership, knowledge of system-specific effectiveness, and experience with the evolving field of healthcare and academic medicine at large [52]. Institutions lose the productivity of staff, with an added burden for those remaining and the additional costs for recruiting and training. From a workforce perspective, lack of staff retention represents a loss of investment and potential productivity which is not sustainable long term [52]. In environments that are increasingly resource-poor, this lack of organizational self-awareness and inertia seems a critical and costly oversight [51]. Sustainable networks which offer innovative solutions to connecting professionally and providing career advancement for health researchers are important for maintaining research quality and health outcomes in regional areas.

We acknowledge several limitations in the present study. These include that the findings are from a pilot program with relatively low number of participants. It is a descriptive study which is not controlled and may therefore be subject to bias. This is mostly likely selection bias due to the selection of mentees from a small pool of healthcare professionals that may not be representative of the sector. Additionally, the formative evaluation collected data which required that mentees recal long periods of time (approximately 10–12 months) and their responses may therefore suffer from recall bias. We did not conduct in depth interviews as part of the formative evaluation and were therefore limited in gaining detailed insight of impact. Future evaluations of this Program and other similar programs should consider investing resources in conducting in depth interviews to assess meeting Program objectives and outcomes.

Despite these limitations, the current study provides tangible measures of outcomes both for the health research sector and those wanting to develop mentoring programs within academic institutions and/ or resource-poor settings.

## Conclusions

This Program has shown promise in providing an interdisciplinary environment and professional networking to specifically deal with aspects of women’s career development, job satisfaction and regional workforce retention. In the future, designing programs that deliberately engage with the corporate sector; that actively support peer-peer mentoring, and offer distance mentoring (i.e. virtual) would be beneficial. In this cohort, 20% of the mentees were off-site and attended all workshops via video conferencing. Given the current travel and meeting restrictions due to COVID-19, we would envisage that virtual workshops and distance mentoring may be an effective conduit to maintain momentum and career progression during such challenging times.

## Supplementary information

**Additional file 1: Table S1.** Needs assessment questionnaire. **Table S2.** Catalyse Mentorship Program Workshops 1–5. **Table S3.** Evaluation questionnaires for mentors. **Table S4.** Evaluation questionnaires for mentees.

## Data Availability

All data generated or analyzed during this study are included in this published article [and its supplementary information files].

## Abbreviations

AUD: Australian dollar
WITH: Women in Tropical Health
