# Supplementary material for: Championing women working in health across regional and rural Australia – a new dual-mentorship model
Source: BMC Med Educ. 2020 Sep 11;20:299. doi: 10.1186/s12909-020-02219-w (PMC7483507; doi:10.1186/s12909-020-02219-w)
Supplement: Supplementary file 1 — Additional file 1: Table S1. Needs assessment questionnaire. Table S2. Catalyse Mentorship Program Workshops 1–5. Table S3. Evaluation questionnaires for mentors. Table S4. Evaluation questionnaires for mentees. [file 12909_2020_2219_MOESM1_ESM.docx]

Supplement

Table S1. Needs assessment questionnaire

| Question |
| --- |
| 1. What do you understand by the term mentoring? 2. A mentee discussing personal and professional goals with a mentor 3. A mentee being coached on a particular task (e.g. grant applications, career progression) 4. Performance appraisal 5. Counselling 6. Other, please specify |
| 1. At what stage in your career do you consider yourself? 2. Early 3. Middle 4. Leadership |
| 1. If a mentoring program were established, would you be interested in taking part? 2. No 3. Yes, because I would like to have access to male and female mentors in diverse roles 4. Yes, because I would like to have access to male and female mentors in different institutes or organisations 5. Yes, for other reasons |
| 1. If a mentoring program were established, would you prefer to choose your own mentor, or to have one allocated as part of a mentor matching process? 2. Choose own mentor 3. Have a mentor allocated to me 4. Don’t mind |
| 1. If a mentoring program were established, would you prefer that the mentee selects you as their mentor themselves, or that one is selected for you as part of a mentor matching process? 2. Mentee chooses me themselves 3. Have a mentee allocated to me 4. Don’t mind |
| 1. Do you currently have a mentor? Or have you previously had one? 2. No 3. Yes, please explain what aspects you found useful |
| 1. Are you currently a mentor? Or previously been a mentor? 2. No 3. Yes, please explain what aspects you found useful |
| 1. Do you have access to a mentoring program/ initiative at your place of work or study? 2. No 3. Yes, could you share your experience |

Table S2. Catalyse Mentorship Program Workshops 1- 5.

| **Workshops** | **Focus** | **Topics** |
| --- | --- | --- |
| Workshop 1 | **“What do you want?”**  Identifying/clarifying what each participant is trying to achieve in line with their personal values and purpose | Why invest in this program?  Priority management  (Covey Quadrant)  White space  Personal Mission Statement Development  Personal Strengths/Weaknesses/Opportunities/Threats Analysis  Bio /Promo/Personal Brand Development  Mentor Acquisition Strategy  Are you ‘Mentorable’? |
| **Mentor Recruitment (Mentor Package) and Mentee Selection** | | |
| Workshop 2 | “How do you get it”  Acquisition strategy workshop including human and financial resourcing | Developing your Tribe (confidant, conduit, connector, advisor, coach)  Emotional Quotient and Social Skills Training  How to talk to a mentor  Meeting outline and preparation  Preparing your ’narrative’  Asking the right questions  Identifying current roadblocks and challenges |
| **Meeting Preparation (i.e. asking the right questions)** | | |
| **Meeting 1** | | |
| Workshop 3 | “How is it going?”  Evaluation and review of mentoring meetings and learnings | Meeting one debrief and learnings  Next Steps Action Plan (Specific Measurable Achievable Realistic Timely Goal Setting)  Creating Powerful Conversations |
| **Meetings 2 and 3** | | |
| Workshop 4 | “How is it going?”  Evaluation and review of mentoring meetings and learnings | Meeting 2 and 3 debrief and learnings  Goals Evaluation  Next steps |
| **Meeting 4** | | |
| Workshop 5 | “What now?”  Strategies to continue to build your network | Personal Mission Statement Review  Personal Development Cycle  The Art of Asking  Evaluation and Next Steps |

Table S3. Evaluation questionnaires for mentors

| **Evaluation Question - Mentors** |
| --- |
| 1. Have you participated in a professional mentorship program before? 2. If yes, please provide details of the program |
| 1. How would you rate the usefulness of the resource pack provided at the start of the program in assisting you to understand your role as a mentor and what to expect from the program?    1. Extremely useful    2. Very useful    3. Somewhat useful    4. Not so useful    5. Not at all useful |
| 1. How useful was your mentee’s personal statement, bio and personal mission statement?    1. Extremely useful    2. Very useful    3. Somewhat useful    4. Not so useful    5. Not at all useful |
| 1. Did these documents help you understand the desired outcomes of your mentee? |
| 1. Would you agree that the matching process used in the Catalyse mentorship program appropriate?    1. Strongly agree    2. Agree    3. Neither agree nor disagree    4. Disagree    5. Strongly disagree |
| 1. How did you find the duration of the program?    1. Too short    2. About the right length    3. Too long |
| 1. How many meetings did you have with your mentee? |
| 1. Did you have any difficulties scheduling meetings with your mentee? 2. If yes, please provide a reason |
| 1. Did you and your mentee stick to the recommended meeting schedule? 2. If no, please provide a reason |
| 1. What were the most common topics of discussion with your mentee?    1. Fellowship applications    2. Promotions    3. Grant writing    4. Work/life balance    5. Career planning    6. Publication Preparation    7. Networking    8. Other: |
| 1. Do you feel that your mentee has achieved the goals set at the beginning of the program? |
| 1. Were there any unexpected outcomes from your participation in the program? |
| 1. Do you feel you received adequate support from the Catalyse program manager? |
| 1. Would you recommend the program to others? |
| 1. Are there any changes you recommend making to any aspect of the Catalyse program? |

Table S4. Evaluation questionnaires for mentees

| **Evaluation Question - Mentee** |
| --- |
| 1. Have you participated in a professional mentorship program before? 2. If yes, please provide details of the program |
| 1. How would you rate the application process for the Catalyse Mentorship Program?    1. The process was very difficult    2. The process was somewhat difficult    3. The process was neither difficult or easy    4. The process was easy    5. The process was very easy |
| 1. How would you rate the usefulness of the resource pack provided at the start of the program in assisting you to understand your role as a mentee and what to expect from the program?    1. Extremely useful    2. Very useful    3. Somewhat useful    4. Not so useful    5. Not at all useful |
| 1. How enjoyable did you find the process of developing your personal brand statement, bio and personal mission statement?   1 2 3 4 5  not enjoyable at all very enjoyable |
| 1. Would you agree that the matching process used in the Catalyse mentorship program appropriate?    1. Strongly agree    2. Agree    3. Neither agree nor disagree    4. Disagree    5. Strongly disagree |
| 1. How did you find the duration of the program?    1. Too short    2. About the right length    3. Too long |
| 1. How did you feel about the number of workshops included in the program?    1. Too many    2. About the right amount    3. Too few |
| 1. Overall, how useful did you find the workshop sessions?    1. Extremely useful    2. Very useful    3. Somewhat useful    4. Not so useful    5. Not at all useful |
| 1. Were there any sessions which you found particularly useful/unuseful? |
| 1. How much of the $2000 bursary did you spend? |
| 1. Please provide details about what you spent your bursary on |
| 1. How useful was the bursary?    1. Extremely useful    2. Very useful    3. Somewhat useful    4. Not so useful    5. Not at all useful |
| 1. How much of an incentive to apply for the program was the inclusion of the bursary?    1. A great deal    2. A lot    3. A moderate amount    4. A little    5. None at all |
| 1. How useful was it having both an academic and a corporate mentor?    1. Extremely useful    2. Very useful    3. Somewhat useful    4. Not so useful    5. Not useful at all |
| 1. If the program offered only one mentor, would you prefer an academic or a corporate mentor? 2. Please provide a reason for this choice |
| 1. How many meetings did you have with your academic mentor? |
| 1. Did you have any difficulties scheduling meetings with your academic mentor? 2. If yes, please provide a reason |
| 1. Did you and your academic mentor stick to the recommended meeting schedule? 2. If no, please provide a reason |
| 1. What were the most common topics of discussion with your academic mentor?    1. Fellowship applications    2. Promotions    3. Grant writing    4. Work/life balance    5. Career planning    6. Publication Preparation    7. Networking    8. Other: |
| 1. How many meetings did you have with your corporate mentor? |
| 1. Did you have any difficulties scheduling meetings with your corporate mentor? 2. if yes, please provide a reason |
| 1. Did you and your corporate mentor stick to the recommended meeting schedule? 2. If no, please provide a reason |
| 1. What were the most common topics of discussion with your corporate mentor?    1. Fellowship applications    2. Promotions    3. Grant writing    4. Work / life balance    5. Career planning    6. Publication preparation    7. Networking    8. Other: |
| 1. What 3 goals did you set at the beginning of the program? |
| 1. Overall, how well do you feel that you have achieved the goals listed above? (1 = very well, 5 = very poorly) |
| 1. Please indicate whether the following have increased after your participation in the program    1. Feeling of interpersonal connectedness in the workplace    2. Job Satisfaction    3. Technical Skills    4. Career opportunities |
| 1. Were there any unexpected outcomes from your participation in the program? |
| 1. Would you recommend the program to others? |
| 1. Are there any changes you recommend making to any aspect of the Catalyse program? |
